# Integrating an ecological approach into an Aboriginal community-based chronic disease prevention program: a longitudinal process evaluation

**DOI:** 10.1186/1471-2458-11-299

**Published:** 2011-05-11

**Authors:** Margaret Cargo, Elisabeth Marks, Julie Brimblecombe, Maria Scarlett, Elaine Maypilama, Joanne Garnggulkpuy Dhurrkay, Mark Daniel

**Affiliations:** 1 Social Epidemiology and Evaluation Research Group, Sansom Institute for Health Research, University of South Australia, City East Campus, Adelaide, 5001, Australia; 2 Centre Hospitalier de l'Université de Montréal, Université de Montréal, 3875 Rue St. Urbain, Montréal, H2W 1V1, Canada; 3 Menzies School of Health Research, Charles Darwin University, John Matthews Building, Royal Darwin Hospital, Rocklands Drive, Casuarina, 0810, Australia; 4 Yalu'Marnggithinyaraw, Galiwin'ku, Elcho Island, 0822, Australia

## Abstract

**Background:**

Public health promotes an ecological approach to chronic disease prevention, however, little research has been conducted to assess the integration of an ecological approach in community-based prevention programs. This study sought to contribute to the evidence base by assessing the extent to which an ecological approach was integrated into an Aboriginal community-based cardiovascular disease (CVD) and type 2 diabetes prevention program, across three-intervention years.

**Methods:**

Activity implementation forms were completed by interview with implementers and participant observation across three intervention years. A standardised ecological coding procedure was applied to assess participant recruitment settings, intervention targets, intervention strategy types, extent of ecologicalness and organisational partnering. Inter-rater reliability for two coders was assessed at Kappa = 0.76 (p < .0.001), 95% CI (0.58, 0.94).

**Results:**

215 activities were implemented across three intervention years by the health program (HP) with some activities implemented in multiple years. Participants were recruited most frequently through organisational settings in years 1 and 2, and organisational and community settings in year 3. The most commonly utilised intervention targets were the individual (IND) as a direct target, and interpersonal (INT) and organisational (ORG) environments as indirect targets; policy (POL), and community (COM) were targeted least. Direct (HP→ IND) and indirect intervention strategies (i.e., HP→ INT→ IND, HP→ POL → IND) were used most often; networking strategies, which link at least two targets (i.e., HP→[ORG-ORG]→IND), were used the least. The program did not become more ecological over time.

**Conclusions:**

The quantity of activities with IND, INT and ORG targets and the proportion of participants recruited through informal cultural networking demonstrate community commitment to prevention. Integration of an ecological approach would have been facilitated by greater inter-organisational collaboration and centralised planning. The upfront time required for community stakeholders to develop their capacity to mobilise around chronic disease is at odds with short-term funding cycles that emphasise organisational accountability.

## Background

To improve health of Australians the new public health calls for action on the ecological determinants of health [1]. Following these calls, public health practitioners have been encouraged to apply an ecological approach to promote active living [2] and to prevent obesity and the development of related chronic diseases such as type 2 diabetes and cardiovascular disease [3].

An ecological approach views health as a product of individuals interacting with their social, physical and cultural environments [4] and seeks to improve health by implementing strategies aimed at modifying the environment and the individual [5]. Environment-focused strategies modify one or more aspects of a priority population's social, political and/or physical environment. One example is schools establishing a school garden to increase the availability of fruits and vegetables for classroom snacks and for the canteen to sell healthful foods. Person-focused strategies engage the priority population in activities that modify their knowledge, attitudes or skills related to one or more behavioural risk factors like, for example, teachers providing students with information on healthful foods. Evidence suggests that ecological interventions can positively contribute to improvements in physical activity behaviour [6], plasma glucose and triglycerides [7], impaired glucose tolerance, hypercholesterolemia, and smoking [8] as well as obesity [6]. Ecological interventions, however, are highly heterogeneous. The ecological complexity of such intervention programs varies according to the settings from which participants are recruited and the combination of individual, interpersonal, community, organisational and policy targets included in the intervention strategies.

A process evaluation procedure has been developed to operationalise the ecological complexity of public health programs[9]. Available research on the application of this procedure suggests that practitioners integrate an ecological approach into their practice, but in so doing, tend to target policy and community environments least often [9-12]. It has also been found that inter-agency collaboration can additionally facilitate the integration of an ecological approach into public health practice[13]. Fundamentally, the formation of partnerships that underpin community mobilisation efforts takes time [14,15]. Given this, one might expect that community-based disease prevention efforts would lead to the implementation of more ecological programs over time as the financial, material and in-kind resources and expertise combined by stakeholders enable health issues to be addressed in ways that each partner could not otherwise achieve on their own. The "collaborative advantage" [16,17] that accrues from the exchange of knowledge and resources between stakeholders should result in ecological programs that reach participants in a variety of settings and which employ a range of educational and environmental change strategies, including policy and community targets.

No study published thus far has prospectively assessed the integration of an ecological approach across three consecutive intervention years, nor has any study done so in the context of an Aboriginal community mobilisation effort for CVD and type 2 diabetes prevention. Here, we hypothesized: (1) that the intervention would become more ecological across the three intervention years; and (2) that a greater proportion of organisations would collaborate in program planning and implementation across the three intervention years. This article reports on longitudinal process evaluation findings.

## Methods

### Setting

The community setting for this healthy lifestyle intervention program was a remote multilingual Aboriginal community (over 20 language groups) of an approximate population of 2,500 in Northeast Arnhemland, Australia. Access to the community is restricted: permission to visit is required by law. Air- and water-based travel between the community and mainland locations is costly. The largest source of income is derived from government payments, although many community members acquire paid employment through a Community Development Employment Program (CDEP). The community was established by the Methodist Overseas Mission in the early 1940's and, while experiencing influences of westernisation since then, community members have retained traditional cultural practices. One of the most pressing issues for community organisations has been the retention of culture for future generations in the face of westernisation, but overall community life remains rooted in traditional culture[18]. The community has diverse services including cultural, educational, recreational, and health services provision.

### The Healthy Lifestyles Project

The Healthy Lifestyles Project was initiated as a participatory research project [19] between the target community and Menzies School of Health Research in response to a community-wide concern about the escalating burden of chronic disease. A planning committee for the Healthy Lifestyles Project, representing various community groups and agencies, formed in July 2001. This led to the voluntary screening of community members aged 15 years and older for type 2 diabetes and cardiovascular risk factors [20]. Screening was completed in March 2002. Community-wide feedback and discussion of screening results was then used as the basis for developing and implementing intervention strategies.

To prevent CVD and the development of type 2 diabetes, the Healthy Lifestyles Project actively promoted a healthful diet, physical activity and smoking cessation and prevention. Part of the original intent of the project was to activate and enable a coordinated community-directed approach to increase the allocation of community resources to prevention activities. Existing community initiatives that supported these healthy lifestyle messages were identified. The aim was to build and support these initiatives by strengthening inter-organisational linkages. Many community organisations and agencies became involved in planning and implementing activities that advanced project goals.

### Data Collection

Prevention activities implemented in the first three intervention years of the Healthy Lifestyles Project (between January 2002 and January 2005) were examined. A two-page activity monitoring form aided collection of data on intervention activities at regular intervals. Forms were completed through a researcher-assisted interview and participant observation process. Open-ended questions were used to obtain information on activity objectives, an activity description, participant recruitment, and to identify organisations that were taking a primary role in decision making related to activity planning and implementation. Given the decentralised approach to CVD and type 2 diabetes prevention programming in the community, interviews were also conducted with members of diverse organisations and professionals working in the local health centre and other agencies. Interviews commenced with health centre staff and representatives of community organisations and government agencies; other organisations and groups involved in implementing healthy lifestyle interventions were identified through a snowball sampling approach which continued until no new organisations implementing healthy lifestyle activities could be identified. Some interviews were done in small sharing circles with 3-4 representatives of the same organisation. This information was translated onto each activity implementation form during the course of the interview. The interviewer's interpretation of the information was verified by those interviewed - either verbally or by reviewing a hard copy of the monitoring form. Data were collected from approximately 30 community stakeholders. Documents such as organisational reports, community newspapers, and program materials were collected to identify activities that may have been missed (resulting in follow-up interviews to properly complete activity implementation forms) and to verify information for those activities already identified. Strong social networks between community members and researchers facilitated the identification of relevant activities.

The protocol was reviewed and clearance provided by the Human Research Ethics Committee of the Northern Territory Department of Health and Community Services and Menzies School of Health Research.

### Coding Scheme

Following the ecological coding scheme developed by Richard [9,12] and refined by Lévesque [10], information obtained on the activity description and objectives was used to code for intervention *targets *and intervention *strategies*, information on participant recruitment was used to code for intervention *setting*, and the information on organisations was used to develop an index of organisations taking a lead or primary role in activity planning and implementation.

Intervention *targets *refer to the sub-group in the community intended to benefit from the intervention, or for whom health behaviour change was designated. Five types of targets are identified: 1) Individuals (IND), 2) Interpersonal environment (INT), 3) Organisations (ORG), 4) Community (COM), and 5) Political players/systems (POL). In this scheme, the health program is annotated as the HP.

All intervention activities implemented by the HP are directed towards an *ultimate target*, or *IND*. The IND represents the primary individual beneficiary or those ultimately designated for change. Thus, health education activities like making brochures available for community members in health clinics on the health risks of smoking are annotated as HP→IND. To be consistent with the communities' prevention efforts, the Healthy Lifestyles Project designated two ultimate targets for their intervention activities: children and community members aged 15 years and older. However, if a single activity was directed towards both groups, children were coded as the ultimate target. Activities were analysed respecting the ultimate target of each activity.

In the case of an intervention strategy including more than one intervention target, a distinction is made between the ultimate target and *proximal target(s)*. A *proximal target *represents any intermediate entity or entities (i.e., INT, ORG, COM or POL), designated for change through implementation of a given intervention activity. Where there is a proximal target designated for change (X), the intervention pathway is specified as 'indirect' and intervenes on the ultimate target through another medium (e.g., HP→ X → IND). For example, children's eating habits at home (IND) can be influenced by engaging mothers in workshops to build their knowledge and skills to purchase healthy food at the grocery store (INT). This, then, gives HP→INT→IND. A networking strategy involves the linking of at least two targets by the program team (HP→[X-X]→IND). One such example is bringing together organisations in a coalition to create a bike path to benefit school children (HP→[ORG-ORG]→IND) [10].

The overall intervention *strategy *represents the sequencing of one or more targets joined either in a direct transformation relationship (i.e., direct transfer of information or resources to the intended target) or in an indirect relationship (i.e., linking at least two targets). Intervention strategies are aggregated into three categories of activities: 1) traditional health education (HP→IND); 2) networking (HP→[X-X]→IND); and 3) indirect transformation (HP→X→IND).

The intervention *setting *is defined as the social system(s) in which persons/entities designated for change are reached. Four types of settings were designated in the coding procedure: 1) Organisation, 2) Community, 3) Society (i.e., state/territory or nation) and 4) Supra-national (i.e., link of two or more societies). Since the traditional extended family structure is strong in this community and a potentially important mechanism for reaching community participants, "Family" was added as a fifth setting for coding activities.

The community organisations, institutions and agencies taking a primary or lead role in activity planning and implementation were identified and listed on the activity implementation form. This enabled determination of the number of lead organisations per activity.

### Analytic Procedure

The analytic phase commenced with the training of two raters in the ecological coding procedure. Information on the activity monitoring forms was coded independently by both raters. Inter-rater agreement was estimated by coding a random sample of 25 activities. Inter-rater reliability was found to be Kappa = 0.76 (p < .0.001), 95% CI (0.58, 0.94). During the coding process, disagreements were noted and resolved through discussion. Frequencies of intervention strategies, intervention settings and intervention strategy types were assessed by year and for differences across the three intervention years. In addition, each organisation was given a score from 0 to 4, with higher scores indicating higher ecological complexity, based on the algorithm developed by Richard [9,12]. A score of 0 was given to an organisation that employed only one intervention strategy, independent of setting and type of strategy. A score of 1 was given to an organisation that employed at least two intervention strategies that did not include HP→IND, regardless of the number of settings in which the strategies were implemented. Scores of 2, 3, and 4 were given to organisations employing an HP→IND intervention strategy and at least one other intervention strategy within 1, 2, and 3+ settings, respectively. The number of lead organisations was assessed by year and across the intervention years as well. PEPI (Version 4) and SPSS (Version 13.0) software were used for descriptive and chi-square analyses. Statistical significance was set at 0.05.

## Results

Data on 131 discrete activities were collected over the three-year Healthy Lifestyle Project; some activities were implemented in more than one year. Analysing these by implementation year, and including repeated activities, there were 215 activities overall. In each of the first three years, 84, 59 and 72 activities were implemented respectively. Significantly more activities were implemented in Year 1 compared to Year 2 (*p *< 0.05). No other comparisons by implementation year were statistically significant.

### Intervention Strategies (Targets)

Table 1 shows intervention activity targets by year. For each year, there was a significant difference in the numbers of activities that targeted the various groups. The most common targets were IND as a direct target and INT and ORG as indirect targets while POL and COM were targeted the least often across all three intervention years. As illustrated in Table 2, eleven intervention strategies were utilised in the Healthy Lifestyles Project. HP→IND was the most common strategy implemented, followed by HP→INT→IND and HP→ORG →IND.

**Table 1:** Frequency of Intervention Targets by Intervention Year

**Type of Target**^**a**^	**Year 1**^**1**^	**Year 2**^**2**^	**Year 3**^**3**^
a. IND as direct target	28 (31%)	17 (26%)	28 (36%)
b. INT	27 (30%)	24 (37%)	23 (30%)
c. ORG	29 (32%)	13 (20%)	15 (19%)
d. POL	3 (3%)	3 (5%)	5 (6%)
e. COM	3 (3%)	8 (12%)	6 (8%)
Total	90	65	77
*Chi-square *(χ^2 ^)_*df = 4*_	41.8	20.2	28.8
*p *value	<0.0001	<0.001	<0.0001

^a^Because a program may contain more than one target, the total frequency exceeds the number of activities.

The following differences in proportions are statistically significant:

^1 ^a-d,e, and b-d,e, and c-d,e *p *< 0.0001

^2 ^a-d and b-e, and c-d *p *< 0.05; b-d *p *< 0.0001

^3 ^a-c and c-d *p *< 0.05; b-e *p *< 0.001; a-d,e, and b-d *p *< 0.0001

**Table 2:** Frequency of Intervention Strategies (with examples) by Intervention Year

Intervention Strategy	Y1	Y2	Y3	Total	Example
1. HP→IND	27	17	27	71	Screening result booklets were returned to community members and results explained.

2. HP→INT→IND	20	18	18	56	Family Food Garden (Burwungatha djama) Project aimed to increase fresh fruit and vegetable consumption. Staff work with families to establish backyard gardens.

3. HP→ORG→IND	16	7	10	33	A new school canteen with good facilities allows nutritious snacks to be prepared and sold to young people, at recess and lunch.

4. HP→COM→IND	2	7	6	15	A community market was informally organised with a variety of market stalls to increase community member's access to fresh fruits, bush foods, fish and shell-fish.

5. HP→[ORG-ORG]→IND	9	3	1	13	A workshop was organised to discuss community screening results. Organisational representatives came together to brainstorm interventions.

6. HP→[INT-INT]→IND	4	3	4	11	A weekly walking program was organised. Families met at the church and walked in the community to have fun and share.

7. HP→ POL→ORG→IND	3	3	4	10	After continuous lobbying and advocacy including to the council, the takeaway manager offered healthier food and kept the deep-fryers off until at least 11 am.

8. HP→[IND-IND]	1	0	1	2	Over 12 teams participate in a community football league, comprised primarily of younger-aged men.

9. HP→[COM-COM]→INT→IND	1	1	0	2	Community members and visitors from surrounding communities receive healthy lifestyle information during a four-day healthy lifestyle festival. Families celebrate relationships and culture.

10. HP→[ORG-ORG]→INT→IND	1	0	0	1	The Commonwealth Nutrition Initiative funded the Health Centre to implement a healthy breakfast program for children. This activity was delivered from the Women's Centre and was supported by community council. It was designed to engage parents in preparing breakfasts as part of school activities.

11. HP→POL→IND	0	0	1	1	Research institute project officers with community researchers lobby with NT Parliamentarians and training providers for funding and political support.

Intervention strategies were aggregated according to direct (HP→IND), indirect (HP→X→IND) or networking (HP→[X-X]→IND) type. Results show statistically significant differences in the type of strategy utilised for each intervention year (Table 3). Networking strategies in the planning and implementation of intervention activities were utilised the least.

**Table 3:** Intervention Strategy Types by Intervention Year

**Intervention strategy type**^**a**^	**Year 1**^**1**^	**Year 2**^**2**^	**Year 3**^**3**^
a. HP→IND	27 (32%)	17 (29%)	27 (38%)
b. HP→X→IND	41 (48%)	35 (59%)	39 (54%)
c. HP→[X-X]→IND	16 (19%)	7 (12%)	6 (8%)
Total	84	59	72
*Chi-square *(χ^2 ^)_*df = 2*_	11.2	20.5	23.2
*p value*	<0.01	<0.0001	<0.0001

^a^Includes repeated activities across intervention years

The following differences in proportions are statistically significant:

^1 ^a-b *p *< 0.05 and b-c *p *< 0.05 and b-c *p *< 0.0001

^2 ^a-c *p *< 0.05 and a-b *p *< 0.01 and b-c *p *< 0.0001

^3 ^a-c, b-c *p *< 0.0001

### Intervention Setting

For any given intervention year, there were statistically significant differences in the settings in which participants were recruited into the Healthy Lifestyles Project. As shown in Table 4, participants were most commonly recruited through organisations (e.g., school, health centre) in Year 1 (χ^2 ^= 67.6_*df = 3*_, *p *< 0.0001) and Year 2 (χ^2 ^= 38.9_*df = 3 *_*p *< 0.0001) and then the community and family settings. Although recruitment differences persisted across settings for Year 3 (χ^2 ^= 35.0_*df = 3*_, *p *< 0.0001), the difference in proportions of participants recruited through organisations and the community setting was not statistically significant. Data were missing for 2 percent of activities.

**Table 4:** Setting for Participant Recruitment by Intervention Year

Setting	**Year 1**^**1**^	**Year 2**^**2**^	**Year 3**^**3**^
a. Organisation	48 (58%)	33 (58%)	35 (49%)
b. Community	28 (34%)	14 (25%)	27 (38%)
c. Family	6 (7%)	9 (16%)	7 (10%)
d. Society	1 (1%)	1 (2%)	2 (3%)
Total	83	57	71
*Chi-square *(χ^2 ^)_*df = 3*_	67.6	38.9	35.0
*p *value	<0.0001	<0.0001	<0.0001

^a^Because a program may contain more than one target, the total frequency exceeds the number of activities. The following differences in proportions are statistically significant:

^1 ^a-b *p *< 0.05 and a-c,d *p *< 0.001

^2 ^c-d *p *< 0.05; a-b and b-d *p *< 0.001 and a-c,d *p *< 0.0001

^3 ^a-c,d and b-c,d *p *< 0.0001

### Lead Organisations

Twenty different organisations, institutions and agencies were involved in leading the planning and implementation of one or more intervention activities across the three-year intervention period. Of the 131 discrete activities, the most frequent leading organisations were the health centre (n = 41 activities), a health research institute (n = 33 activities), a cultural organisation led solely by community members (n = 15 activities) and the local education centre (n = 11 activities). The associated health research institution partnered with the cultural organisation in all activities that the latter body initiated. The remaining 16 organisations represented community organisations, and educational and government agencies. Based on all 215 activities (Table 5), the proportions of activities implemented by the partnering of two or more lead organisations were similar for Year 1 (13 percent) and Year 2 (12 percent). The proportions of activities implemented by organisational partnering significantly increased in Year 3 (27 percent) (χ^2 ^= 6.3_*df = 2 *_*p *< 0.05). The difference in proportions between Year 1 and 3 was statistically significant (*p *< 0.05), with a trend towards significance between Year 2 and 3 (*p *= 0.07).

**Table 5:** Number of Lead Organisations by Intervention Year

No. Lead Organisations	Year 1 (n/%)	Year 2 (n/%)	Year 3 (n/%)	***Chi-square *(χ**^**2 **^**)**
1	71 (87%)	51 (88%)	52 (73%)	6.3_*df = 2*_
2 +	11 (13%)	7 (12%)	19 (26%)	*p *< 0.05

### Ecological Approach

Organisations received a score representing the ecological complexity of intervention activities for which they were designated as lead organisations (applying the algorithm described earlier). As shown in Table 6, there was no significant difference in mean ecological complexity scores in the three intervention years. It should be noted, however, that the four organisations with a health mandate had numerically higher ecological scores.

**Table 6:** Ecological Scores for Organisations Implementing Activities by Intervention Year

**Ecological Score**^**a**^	Year 1	Year 2	Year 3
0	8	10	12
1	3	1	2
2	3	4	4
3	3	2	1
4	0	0	1
Total	17	17	20
Mean (95% CI)	1.06 (0.44-1.67)	0.88 (0.28-1.48)	0.85 (0.28-1.42)

^a^Ecological scores are given only for organisations that implemented activities during the given implementation year.

Ecological mean scores are not statistically different; *p *> 0.05

## Discussion

Community members and service providers residing in this remote Aboriginal community, in addition to the external partnering research institution, implemented 215 activities across three intervention years. This level of implementation demonstrates a high degree of community commitment to prevention of CVD and type 2 diabetes. With respect to the hypotheses, although the Healthy Lifestyles Project did not become more ecological over time (hypothesis 1) there was some evidence to support greater organisational partnering over the three-year project (hypothesis 2). These findings, reviewed below, should be interpreted in light of barriers experienced to implementation, and logistical and measurement challenges related to data collection and analyses.

### Intervention strategies, settings and ecologicalness

Similar to other intervention studies applying the ecological coding procedure [10-12,21] the majority of activities targeted by the Healthy Lifestyles Project were of the form IND, INT, or ORG. Community champions found activities with these targets necessary for raising the community's consciousness of the risk factors for CVD and type 2 diabetes and for motivating other community members to champion the Healthy Lifestyle Project. Indeed, experiences from other Aboriginal communities [22] and the community readiness literature [23,24] suggest that activities with IND, INT and ORG targets may be necessary precursors to shifting a community from 'no awareness' to the 'pre-planning' and 'preparation' stages of readiness for intervention implementation. Future developments of the ecological coding procedure may need to consider the relative timing of implementing particular intervention strategies in relation to the level of community readiness.

Almost all participants were recruited into activities through organisational, family and community settings (via cultural networks and program champions). Integration of an ecological approach would have been strengthened by the introduction of activities that recruited participants from supra-national and societal settings. In this study, no participants were recruited through supra-national settings and few activities recruited participants though a societal setting. Examples of such actions could be local adoption of one or more recommendations from an international physical activity charter ('supranational' setting) or a state/territory policy offering community subsidies to transport fruits and vegetables to remote area grocery stores ('society' setting).

Our findings show that the Healthy Lifestyles Project did not become more ecological over time. We attribute this, in part, to the decentralised activity planning and implementation process. Academic researchers partnered with the community to screen participants for CVD risk factors and type 2 diabetes. Community research assistants collected these data in partnership with academic researchers, who were committed to strengthening community capacity. Results were returned to individual participants with full explanation (and a referral to the clinic, if necessary) and collectively to the community, with the intent of mobilising key stakeholders in an organised planning and implementation process [22,25,26]. Despite a cultural organisation championing the project, no central organising body with community-wide representation was able to form during the three-year intervention period. Consideration of barriers, below, illuminates some of the cultural and community factors that may have conspired against the formation of a centralised process.

### Partnerships

Encouragingly, a greater proportion of activities involving inter-organisational collaboration was implemented in Year 3 as compared to the previous years. This was accounted for through the partnering of two organisations, one of these being the cultural organisation championing the project. Few programs involved more than two organisations in decision-making during the planning and implementation stages of a given intervention activity. Most interventions were primarily planned and implemented by a single organisation. This contrasts with the Kahnawake Schools Diabetes Prevention Project (KSDPP) in which one-half of activities involved partnering by Year 3 [10]. In KSDPP, activities were planned and implemented through a centralised process.

A higher (or better) ecological score depends on organisations applying direct (e.g., IND) and indirect (e.g., INT, ORG) intervention strategies to reach participants in multiple settings (e.g., community, organisation). Had organisations partnered more frequently, their ecological score could potentially have been improved through the "collaborative advantage" accruing from organisations pooling their collective knowledge and resources to further the recruitment of participants across multiple settings and using their collective power to lobby for political (POL) and structural changes (COM, ORG). Such forms of changes are less attainable through the efforts of a single organisation [17]. An excellent example of collaborative advantage comes from advocacy efforts that encouraged a very influential take-away food manager in the community to offer more healthful food options and to keep deep-fryers off until at least 11 am.

The efforts of certain change agents in the community stand out. These change agents were practitioners and cultural leaders dedicated to a holistic approach to health. They collaborated with other organisations and implemented person- and environment-centred strategies while recruiting participants from multiple settings. The organisations of these change agents received the highest ecological scores. In line with research on the ecological approach [27], these practitioners may have perceived themselves as having the skills to develop and/or implement environmental interventions and thus were more likely to target the environment for change than were others in the community.

### Barriers to implementation of an ecological approach

Several factors may have conspired against the integration of a community-directed ecological approach and greater inter-organisational partnering in this remote Aboriginal community. These barriers represent the reflections of the study co-authors, which include two community members.

No single agency in the community was funded or had a mandate to co-ordinate prevention of CVD and type 2 diabetes. Even though a cultural organisation championed the project, it was apparent that some organisations found it difficult to work collaboratively around a shared vision whilst in competition for limited financial resources. Because organisations experienced pressure to demonstrate short-term accountability for deliverables they were less able to invest time and resources to work together to achieve longer-term outcomes required for primary prevention. Given the burden of chronic disease and issues relating to basic services, education, housing, development and social harmony, organisations tended to invest their limited resources in treatment, and respond to health and community crises. The sole organisation that partnered with the associated health research institution, which attempted to take a leadership role in facilitating centralised planning, did so while continually justifying this time investment to their funders.

Some Aboriginal stakeholders were sceptical of projects funded under western systems of accountability. Such projects were perceived not to respect cultural norms of non-interference and obligation. Community members emphasised the importance of non-Aboriginal community members working within established cultural respect frameworks [28]. Conflicts between western and cultural systems acted as barriers to Aboriginal stakeholders forming partnerships. The high turnover of non-Aboriginal staff in organisational management positions also led to fatigue in inter-organisational engagement where partnerships were often formed on a personal rather than organisational level. Contributing to this sometimes tenuous relationship between organisations, were differences between clan groups represented within the different organisations. Further, western funding mechanisms did not consider the upfront time required to develop Aboriginal community member's program planning, management and research capacity, inclusive of understanding of how to influence policy. Capacity development, considered key to Aboriginal self-determination and program sustainability [7,29], would have taken at least two years for the community to mount a coordinated ecological approach to community-based CVD and type 2 diabetes prevention.

Beyond community activation it can take many years for a community to build sufficient capacity to implement policy-level changes that promote program sustainability [30]. As one example, six months after completion of the three-year evaluation of the Healthy Lifestyles Project, the Aboriginal owned store association implemented a Nutrition and Health Strategy in five communities to improve the nutritional quality of each store's food supply. This Strategy built on the three years of community-wide health promotion activity implementation associated with the Healthy Lifestyles Project. Recruiting community members into interventions implemented by the state or commonwealth ("societal") or broader supranational settings was constrained by community remoteness, the use of English as a second or third language, and the upfront time required to develop networks with external Aboriginal and non-Aboriginal stakeholders in the business, government and non-profit sectors. Moreover, the policy-making arena is largely perceived as a foreign process occurring external to the control of Aboriginal community members. Policy-making requires two-way learning whereby Aboriginal community members can share their expertise in grassroots community action and governance processes, while non-Aboriginal stakeholders can offer insights into collaborative planning to influence institutional structures and support policy implementation.

### Study Limitations

Study findings should be interpreted in light of the following logistical and measurement challenges and limitations.

Organisations in this community had previously been exposed to other participatory research projects, but were more accustomed to research focusing on outcomes, rather than process evaluation. Consequently, many organisations placed lesser value on completing activity monitoring forms, necessitating greater research assistant involvement toward ensuring data collection. Given that the community was only accessible by boat or air, considerable advance planning was required for researchers to collect data. Community remoteness also hampered researcher's efforts to develop community capacity through training and provision of ongoing mentoring for community research assistants. These logistical issues were compounded by difficulties in arranging times with implementing organisations and agencies to complete activity monitoring forms which arose, in part, from lack of a community norm for fixed schedules, and unanticipated cultural events and obligations. The persistence of the data collection effort and ongoing support of key community leaders in this study resulted in key findings likely less affected by such types of challenges.

From a measurement perspective, the extent of ecologicalness could have been under-estimated in our analysis. Organisational partnering occurring through culturally embedded collaborative planning could have occurred but may not have been identified on the monitoring forms. The ecological coding procedure may be limited in that equal statistical weighting is given to IND, INT, ORG, COM, POL and networked intervention targets (ie., ORG-ORG). It is the case, however, that significantly more time, collaborative capacity and resources are required to mount such types of activities in comparison to those with direct (HP→ IND) or indirect interpersonal (HP→ INT→ 1ND) targets. Moreover, intervention strategies that include distal POL targets (e.g., HP→ POL → IND) in which participants are recruited from societal or supranational settings carry the same weight as an intervention strategy in which clients are directly targeted (HP→ IND) through a proximal organisational setting. The coding algorithm as it stands may not adequately discriminate between and/or differentially weight such types of intervention strategies, particularly at the low end of the scale. This study has inspired further development of the ecological algorithm.

## Conclusions

Longitudinal process evaluation of this community-based intervention suggests that greater time may be required for Aboriginal communities to mount a coordinated ecological approach for interventions to prevent CVD and type 2 diabetes. To do so, Aboriginal community capacity building is essential to facilitate the implementation of culturally sanctioned change strategies. Future research should seek to understand the resources and supports required to enable Aboriginal communities to mobilise and address their health issues.

## Competing interests

The authors declare that they have no competing interests.

## Authors' contributions

MD and MC designed the study. MC, EMar, EMay, JB, MS, EM, JGD were involved in data collection and verifying activity monitoring forms. MC and EMar analysed the data. MC, EMar, JB and MD made contributions to interpreting the findings and manuscript writing; EMay and JGD were involved in verifying and interpreting the findings, and contributing to the discussion section. All authors read and approved the final manuscript.

## Pre-publication history

The pre-publication history for this paper can be accessed here:

http://www.biomedcentral.com/1471-2458/11/299/prepub
